# Mental Health and Behavior of College Students During the Early Phases of the COVID-19 Pandemic: Longitudinal Smartphone and Ecological Momentary Assessment Study

**DOI:** 10.2196/20185

**Published:** 2020-06-17

**Authors:** Jeremy F Huckins, Alex W daSilva, Weichen Wang, Elin Hedlund, Courtney Rogers, Subigya K Nepal, Jialing Wu, Mikio Obuchi, Eilis I Murphy, Meghan L Meyer, Dylan D Wagner, Paul E Holtzheimer, Andrew T Campbell

**Affiliations:** 1 Department of Psychological and Brain Science Dartmouth College Hanover, NH United States; 2 Department of Computer Science Dartmouth College Hanover, NH United States; 3 Department of Psychology Ohio State University Columbus, OH United States; 4 National Center for PTSD White River Junction, VT United States; 5 Department of Psychiatry Dartmouth-Hitchcock Medical Center Lebanon, NH United States

**Keywords:** COVID-19, depression, anxiety, mobile sensing, sedentary, phone usage, mental health, behavior, pandemic, app

## Abstract

**Background:**

The vast majority of people worldwide have been impacted by coronavirus disease (COVID-19). In addition to the millions of individuals who have been infected with the disease, billions of individuals have been asked or required by local and national governments to change their behavioral patterns. Previous research on epidemics or traumatic events suggests that this can lead to profound behavioral and mental health changes; however, researchers are rarely able to track these changes with frequent, near-real-time sampling or compare their findings to previous years of data for the same individuals.

**Objective:**

By combining mobile phone sensing and self-reported mental health data among college students who have been participating in a longitudinal study for the past 2 years, we sought to answer two overarching questions. First, have the behaviors and mental health of the participants changed in response to the COVID-19 pandemic compared to previous time periods? Second, are these behavior and mental health changes associated with the relative news coverage of COVID-19 in the US media?

**Methods:**

Behaviors such as the number of locations visited, distance traveled, duration of phone usage, number of phone unlocks, sleep duration, and sedentary time were measured using the StudentLife smartphone sensing app. Depression and anxiety were assessed using weekly self-reported ecological momentary assessments of the Patient Health Questionnaire-4. The participants were 217 undergraduate students, with 178 (82.0%) students providing data during the Winter 2020 term. Differences in behaviors and self-reported mental health collected during the Winter 2020 term compared to previous terms in the same cohort were modeled using mixed linear models.

**Results:**

During the first academic term impacted by COVID-19 (Winter 2020), individuals were more sedentary and reported increased anxiety and depression symptoms (*P*<.001) relative to previous academic terms and subsequent academic breaks. Interactions between the Winter 2020 term and the week of the academic term (linear and quadratic) were significant. In a mixed linear model, phone usage, number of locations visited, and week of the term were strongly associated with increased amount of COVID-19–related news. When mental health metrics (eg, depression and anxiety) were added to the previous measures (week of term, number of locations visited, and phone usage), both anxiety (*P*<.001) and depression (*P*=.03) were significantly associated with COVID-19–related news.

**Conclusions:**

Compared with prior academic terms, individuals in the Winter 2020 term were more sedentary, anxious, and depressed. A wide variety of behaviors, including increased phone usage, decreased physical activity, and fewer locations visited, were associated with fluctuations in COVID-19 news reporting. While this large-scale shift in mental health and behavior is unsurprising, its characterization is particularly important to help guide the development of methods to reduce the impact of future catastrophic events on the mental health of the population.

## Introduction

### Coronavirus Disease

An outbreak of severe acute respiratory syndrome coronavirus 2 (SARS-CoV-2), the virus that causes coronavirus disease (COVID-19, also known as 2019-nCov), was first reported in Wuhan, China in December 2019, and SARS-CoV-2 was identified as a novel coronavirus in January 2020. On March 11, 2020, the World Health Organization (WHO) declared COVID-19 a global pandemic; as of April 27, 2020, COVID-19 was responsible for over 200,000 deaths and 3,000,000 confirmed cases worldwide [1]. COVID-19 is not only a grave public health issue; it also carries severe political, economic, educational, and social ramifications. COVID-19 continues to impact millions of people worldwide every day. Understanding the behavioral and mental health implications for individuals during this unprecedented period of high stress and crisis is critical for informing current public policies and ensuring preparedness for future pandemics.

### Mental Health and Behaviors in Pandemics and Disasters

Initial survey-based research on the psychological impact of the COVID-19 outbreak in China suggested that the mental health impact was moderate to severe for the majority of respondents in the general population, with increased anxiety, depression, and stress attributed to the outbreak by participants [2]. In initial research investigating the impact of the COVID-19 outbreak on college students in China, increased levels of anxiety and depression were observed as well as a willingness to engage in social isolation [3,4]. A limitation of these studies is that they used cross-sectional data; better understanding of the timeline of onset of anxious and depressive symptoms is critical if we are to understand how mental health changes in response to different stages of the pandemic (eg, initial reports, the first national infections, and shelter-in-place).

A handful of studies have employed ecological momentary assessments (EMAs) to assess depression and anxiety more frequently and in near-real-time [5-8]. EMA surveys are sent at predetermined frequencies to the participants’ phones as they go about their daily life. This method enables the collection of dense longitudinal data with minimal participant effort relative to in-person research. An initial study in which EMAs from 80 undergraduate students were used to investigate the influence of COVID-19 on mental health and social contact found increased mental health problems but no change in social contact [9].

**Figure 1:**
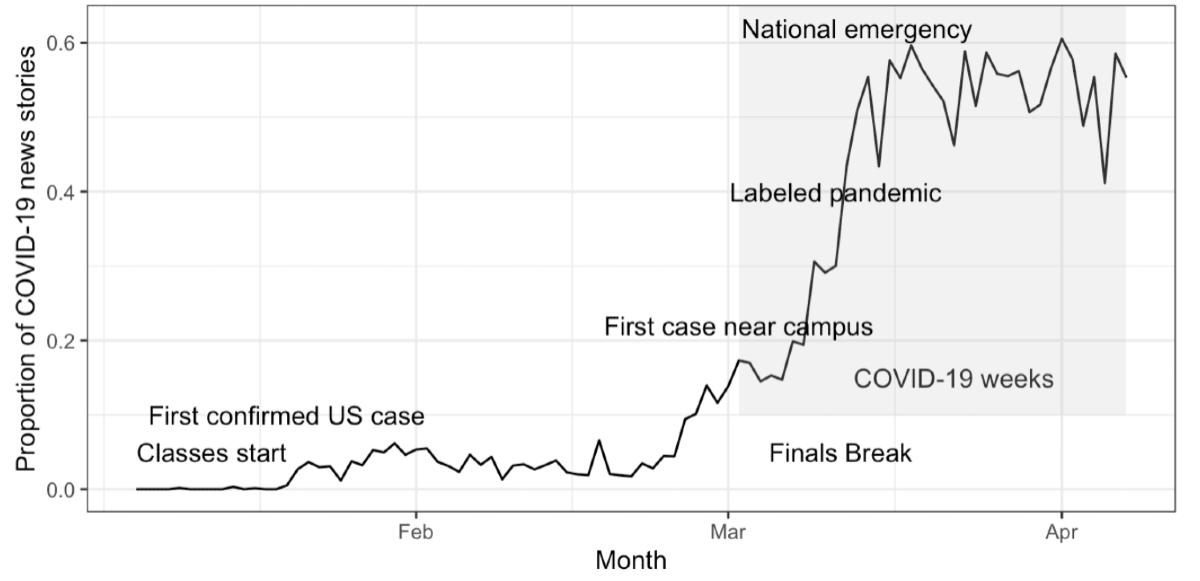
Proportion of US news and media stories containing the term *coronavirus* with key COVID-19 and Dartmouth academic term events labeled. Data were obtained from Media Cloud. COVID-19: coronavirus disease.

Our current work combines longitudinal smartphone sensing and EMAs collected from a cohort of Dartmouth College undergraduates to determine the impact on mental health and behaviors during the COVID-19 pandemic. Dartmouth College has four academic terms per year, which are roughly split into 10-week terms followed by 2-week (or longer) breaks. Self-reported mental health has been observed to vary across typical academic terms [8,10]. During the Winter 2020 academic term, Dartmouth College began implementing new policies in response to COVID-19 (Table 1). Beginning on February 4, individuals returning from China were asked to self-isolate for 14 days upon their return to campus. On March 2, the first local COVID-19 case was diagnosed at nearby Dartmouth-Hitchcock Medical Center. On March 10, during the final examination period, the College requested that all students scheduled to leave campus for spring break depart upon completion of their final examinations and further requested that students who were planning to stay on campus during spring break instead depart campus by March 16. Consequently, students were required to rapidly change their travel plans, complete final examinations sooner than expected, or take final examinations online. On March 11, the WHO officially declared COVID-19 a pandemic, at which time all college-sponsored athletic competitions were cancelled. Just one day later, on March 12, the US government implemented policies limiting travel to and from European countries. By the next day, March 13, COVID-19 had been declared a national emergency by the President of the United States. Dartmouth College’s spring break started on March 14. On March 16, the college cancelled all gatherings of groups larger than 50 people. Finally, on March 17, the college announced that there would be no in-person, on-campus option to attend classes during the Spring 2020 term. This timeline is particularly relevant because it allows us to identify periods during which we might expect additional changes in mental health and behaviors due to the stress of the pandemic and in light of potential adherence to the “Stay Safe, Stay Home” policies mandated by local and national governments. News coverage in the United States can also serve as a proxy for the perceived severity of the situation, given the rapid transition from a localized outbreak in a country several thousand miles away (China) to an outbreak within a few miles of campus along with a rising number of cases nationally (Figure 1). Increases in anxiety and depression are frequently observed after traumatic events [11-14].

**Table 1:** Key Dartmouth College academic dates for the Winter 2020 term and relevant COVID-19 events.

Date	Event	Term week
January 6	First day of classes	1
January 20	First confirmed COVID-19^a^ case in the United States	3
February 4	Travelers from China asked to self-quarantine	5
March 2	First COVID-19 case near campus	9
March 6	Last day of classes	9
March 9	First day of final examinations	10
March 10	Students asked to leave campus as soon as possible (March 16 at the latest)	10
March 11	WHO^b^ labels COVID-19 a pandemic; all Dartmouth College athletics cancelled	10
March 12	Travel between the United States and Europe restricted	10
March 13	COVID-19 declared a national emergency; last day of final examinations	10
March 14	Start of spring break	11 (Break)
March 16	Gatherings of more than 50 individuals cancelled	11 (Break)
March 17	Online-only off-campus learning for Spring 2020 term announced	11 (Break)

^a^COVID-19: coronavirus disease.

^b^WHO: World Health Organization.

### Objectives

The current work seeks to answer the following questions. First, how and when was the mental health of college students impacted by the COVID-19 epidemic, and were changes in depression and anxiety statistically different from those in previous terms in the same student cohort? Second, how are daily behaviors (as measured by smartphone sensing) and changes in depression and anxiety impacted by COVID-19 media coverage?

## Methods

### Study Design

All data in the current study were obtained from the second iteration of the StudentLife study [11], which is a longitudinal multimodal study that is designed to follow the experiences of undergraduate students throughout their academic tenure, with a focus on mental health. Study components include smartphone mobile sensing through the StudentLife app [6], EMAs and surveys focusing on a variety of college experience components, and functional neuroimaging [12].

### Participants

Data were collected from 219 participants who agreed to provide mobile sensing data via the StudentLife app [6]. One participant was removed from the study because their mobile phone was incompatible with the app, and one participant withdrew within a week of starting the study. Data for both subjects were excluded from further analyses. Of the remaining 217 participants, 147 (67.8%) were female, with an age range for all participants of 18 to 22 years at the time of enrollment. Recruitment for this study began in August 2017 and concluded in November 2018. This study was approved by Dartmouth’s Committee for the Protection of Human Subjects.

### Academic Terms

At Dartmouth College, the academic calendar consists of a flexible, year-round calendar that is approximately divided into four academic terms, or quarters. Each term consists of 10 weeks, typically followed by 2 (or more) weeks of break. The Winter 2020 academic term (January 6 start date) includes the progression of the COVID-19 pandemic, ranging from the first confirmed case in the United States (Table 1) to the identification of COVID-19 as a global pandemic; these events resulted in policy and regulatory changes at the college, local, and national levels. Academic terms prior to the Winter 2020 term, including the subsequent 2 weeks of break, were included as control terms.

### Mobile Sensing and EMAs

Smartphone sensing data and EMA surveys were administered using the StudentLife application (iOS and Android) [6]. The StudentLife app collects data from several of the phone’s sensors, including but not limited to the GPS, accelerometer, and lock/unlock status. Anonymized data from the StudentLife app are uploaded to a secure server when a participant is both using WiFi and charging their phone. Data from these sensors are used to assess factors such as the day-to-day and week-by-week impact of workload on the stress, sleep, activity, mood, sociability, mental well-being, and academic performance of the students [6]. Students are prompted weekly by the StudentLife application to complete a few short surveys, administered as EMAs [5]. These EMAs include the Patient Health Questionnaire-4 (PHQ-4), a brief measure of depressive and anxious symptoms [13] that assesses how often individuals were bothered by specific symptoms over the last 2 weeks with values ranging from 0 to 6 for each subscale. The PHQ-4 combines the Patient Health Questionnaire-2 (PHQ-2) and the Generalized Anxiety Disorder-2 (GAD-2). Data coverage was 19.7/24 hours for location, 22.3/24 hours for all other indices, and 80.1% for EMA responses across the duration of the study.

### Sedentary Time

Sedentary time, or stationary duration, is computed to measure students’ activity or, more precisely, their lack of activity. The app continuously infers physical activities using the Android activity recognition application programming interface [14,15] or iOS Core Motion [16].

### Sleep

Sleep was inferred through a combination of passive sensing features (ambient light, movement activity, screen on/off). In this way, 3 features were computed: sleep onset, wake time, and sleep duration. These measures of sleep have been shown to be accurate within 30 minutes for total sleep duration [6].

### Location

Density-based spatial clustering of applications with noise (DBSCAN) [17] was used to cluster GPS coordinates to determine the number of locations visited and distance traveled during a given time period. Locations were detected when 3 GPS samples (1 sample every 10 minutes) were within a radius of 30 meters. Distance was calculated in meters traveled between all locations throughout the day.

### Phone Usage

Unlock duration is a measurement of time during which a mobile phone is unlocked and the screen is on; it is calculated from the time the user unlocks the phone until they either manually relock the phone or it autolocks due to disuse (the iOS default is 30 seconds, while Android defaults vary by manufacturer; users can also alter this by changing their phone settings). Notification and system services do not influence the measurement of unlock duration. While unlock duration is not an absolute measurement of phone usage, it is the closest approximation implemented in StudentLife. From the start of the study in September 2017 until September 2018, the unlock duration was measured by remotely triggering the mobile phones every 10 minutes, sampling 1 minute every 10 minute period (minimum 10% temporal coverage). If conversation was detected during the 1-minute sampling period, the sampling was extended to 3 min for a maximum of 30% temporal coverage. After September 2018, the mobile phones were remotely triggered every 3 minutes, with subsequent sampling for 1 minute. Lock/unlock behaviors within that minute were recorded in real time, while lock/unlocks for the remaining 2 minutes were logged during the next remote trigger.

### COVID-19 News Coverage

To obtain an unbiased measurement of media exposure to COVID-19, the number of news articles published with the term *coronavirus* and the number of all news articles were pulled for the duration of the entire study (August 2017 to 2 weeks after the end of the Winter 2020 academic term). Articles were pulled from a variety of US news outlets, including newspapers and online sources, on the Media Cloud website [18]. The ratio of articles mentioning *coronavirus* to the total number of articles was computed to create a variable indexing the amount of COVID-19 reporting in the media landscape and is presented alongside notable timepoints in the academic term (Figure 1).

### Data Processing, Modeling, and Visualization

**Figure 2:**
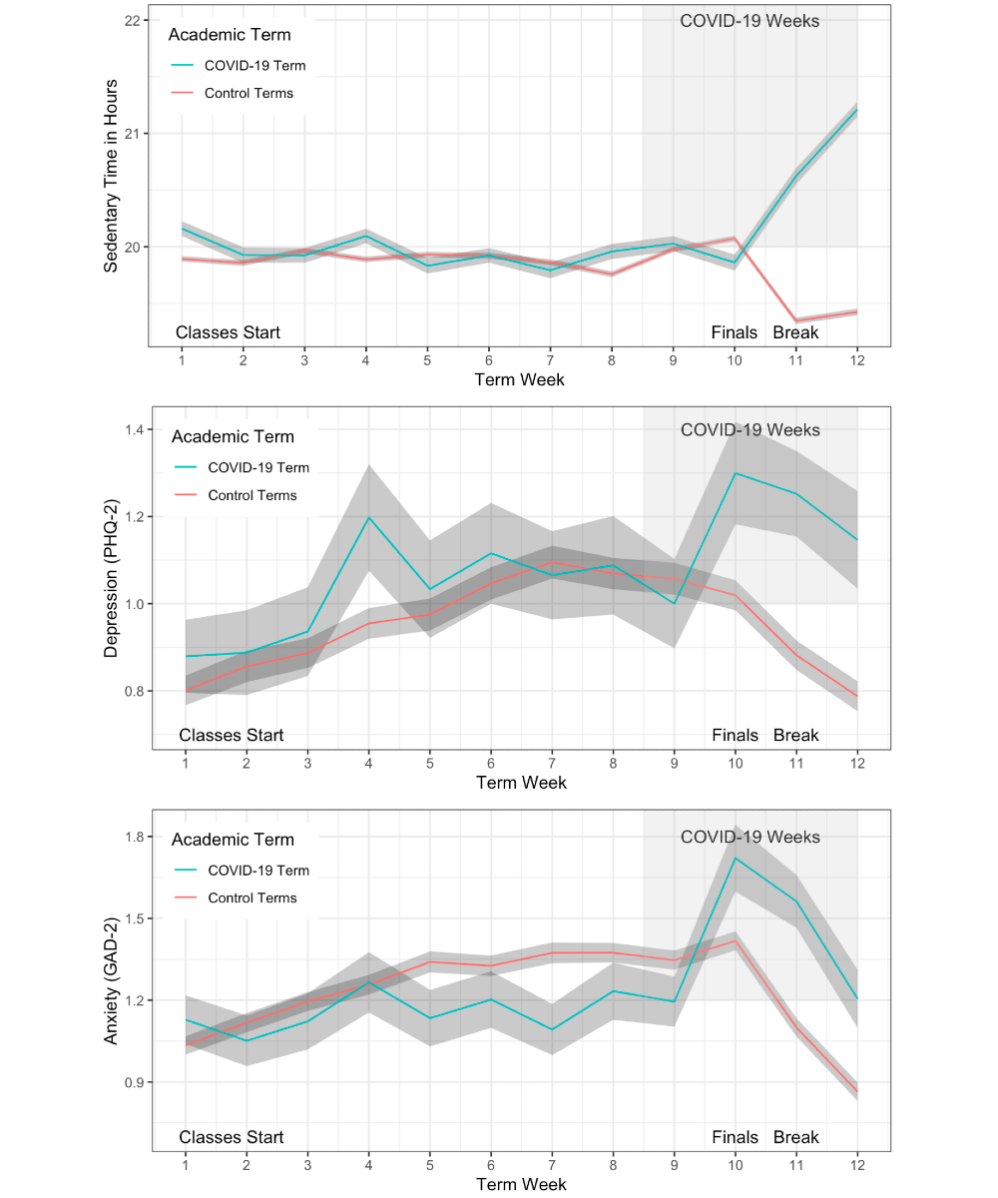
Sedentary time (top), depression (middle), and anxiety (bottom) scores across an academic term and the first 2 weeks of break, with the term influenced by the outbreak of the COVID-19 pandemic as a separate line. The shaded ribbons represent the standard error for each week. Weeks influenced by policy changes related to COVID-19 are represented with a shaded box from weeks 9 to 12. Sedentary time was calculated using data from the StudentLife app. Depression and anxiety were measured with the PHQ-2 and GAD-2 scales through the StudentLife app. Control terms include data from the same group of individuals across previous academic terms. COVID-19: coronavirus disease; Finals: final examinations; GAD-2: Generalized Anxiety Disorder-2; PHQ-2: Patient Health Questionnaire-2.

Data processing was performed in R [19] with formatting and development in R Markdown using RStudio [20]. Modeling was implemented in the lme4 [21] and lmerTest [22] packages. Plots were generated using ggplot2 [23]. Result tables were produced using the stargazer package [24]. The objective of the first analysis was to model sedentary time, depression, and anxiety. To observe trends in these domains across the academic term, the mean values of the variables of interest were plotted by week of the term; data were combined from all study participants from all terms except for the Winter 2020 term, which was plotted as a separate line. Standard error was plotted as a shaded ribbon surrounding the mean. Visual representations of the sedentary time and self-reported depression and anxiety can be observed in Figure 2. Linear mixed models fit by log-likelihood were compared to determine if the values were indeed different from prior terms with respect to sedentary time, anxiety, and depression. The terms in each model included a binary factor, “COVIDTerm,” to label whether the term was influenced by COVID-19. The term week was modeled as linear and quadratic factors, along with interactions between the COVIDTerm factor and the term week variables (ie, COVIDTerm×Term Week (Linear)) in subsequent models. Random intercepts were set per subject in all models. Term week variables were scaled to aid model convergence.

Each variable of interest (sedentary time, depression, and anxiety) was individually modeled with COVIDTerm, term week (linear), and random intercepts per subject. The next model added an interaction term between COVIDTerm and term week (Linear). The third model added the term week (quadratic) variable, and the fourth model added an interaction between COVIDTerm and term week (quadratic). For each of the variables of interest, these four models were compared using the analysis of variance function from the base stats package in R. For each variable, the model with the lowest deviance was selected. *P* values were calculated using the Satterthwaite method as implemented in lmerModLmerTest as part of the lmerTest package.

To obtain a daily variable mirroring possible exposure to COVID-19–related news content, a *coronavirus* topic was created on Media Cloud with dates spanning the duration of the study. The proportion of stories including *coronavirus* was downloaded and scaled. Modeling of COVID-19 news was first performed by combining smartphone sensing features and the week of the academic term. Each feature was scaled before being submitted to the model. Each variable, except for the COVIDTerm factor and subject, was scaled to aid model convergence and allow for regression coefficients that can be compared for relative importance. COVID-19 news was inferred with fixed effects of unlock duration, unlock number, sedentary time, sleep duration, number of locations visited, and term week (linear and quadratic) variables, as well as random intercepts for each subject.

To determine if mental health was associated with the COVID-19 news ratio, self-reported depression and anxiety variables were added to the daily model above. Given that individual participants only answered mental health EMAs on a weekly basis, the subsequent models include far fewer time points than the smartphone-sensing only model above; again, this limits the statistical comparability between the models.

## Results

### Behavioral and Mental Health Changes Associated With the COVID-19 Pandemic

Compared with all other academic terms, we observed differences in both behavior and mental health between weeks 9 and 12 of the Winter 2020 term, which correspond to the last week of classes, the week of final examinations, and the 2-week spring break. Sedentary time appeared to be very similar across the Winter 2020 and previous terms until week 11 (first week of break), when individuals spent over one hour more per day sedentary compared with a typical term; the sedentary time increased further through the second week of break (Figure 2). Self-reported symptoms of depression and anxiety spiked noticeably in week 10, which corresponds to widespread policy changes at the college, local, and national government levels. Week 10 is 1 week after the first case of COVID-19 was confirmed proximal to Dartmouth College, which occurred on the first day of week 9 of the academic term. Week 10 also corresponds to the time when students were asked to leave campus as soon as possible and when the switch to a remote learning model for the Spring 2020 term was announced. After week 10 in the Winter 2020 term, both depression and anxiety remained consistently elevated above the levels in other terms; however, they decreased at similar rates.

Multiple models for sedentary time, anxiety, and depression were tested (see the Methods section for specific details). For each of these variables, superior fit (measured by lowest deviance) was observed with the most complex model. This model included the COVID-19 term, linear term, and quadratic term week trends as well as the interaction of the COVID-19 term with each of the term week trends; the model also allowed for random intercepts for each participant’s data (random effects). Modeling of the academic term affected by COVID-19 compared with academic terms prior to the COVID-19 pandemic identified significantly increased sedentary time, depression, and anxiety (*P*<.001; Table 2). Interactions of the COVID-19 term and quadratic term week regressor for all three variables (*P*<.001) and significant interactions between the COVID-19 term and the linear term week regressor for sedentary time and depression were also observed (*P*<.001 and *P*=.004, respectively).

**Table 2:** Models of sedentary time, depression, and anxiety by week and by presence of COVID-19 during the academic term.

Variable	Dependent variables					
	Sedentary time (observations=113,864)		Depression (observations=20,323)		Anxiety (observations=113,864)	
	Parameter (SD)	*P* value	Parameter (SD)	*P* value	Parameter (SD)	*P* value
COVID-19^a^ term	0.150 (0.008)	<.001	0.176 (0.016)	<.001	0.111 (0.017)	<.001
Term week (linear)	–0.046 (0.003)	<.001	0.016 (0.005)	.004	0.004 (0.006)	.51
Term week (quadratic)	–0.045 (0.003)	<.001	–0.078 (0.006)	<.001	–0.108 (0.006)	<.001
COVID-19 term: term week (linear)	0.138 (0.008)	<.001	0.058 (0.016)	.001	0.079 (0.016)	<.001
COVID-19 term: term week (quadratic)	0.160 (0.008)	<.001	0.064 (0.016)	<.001	0.123 (0.016)	<.001
Constant	–0.040 (0.032)	.21	0.012 (0.046)	.80	0.040 (0.045)	.37

^a^COVID-19: coronavirus disease.

### COVID-19 News Coverage, Mental Health, and Mobile Sensing

**Figure 3:**
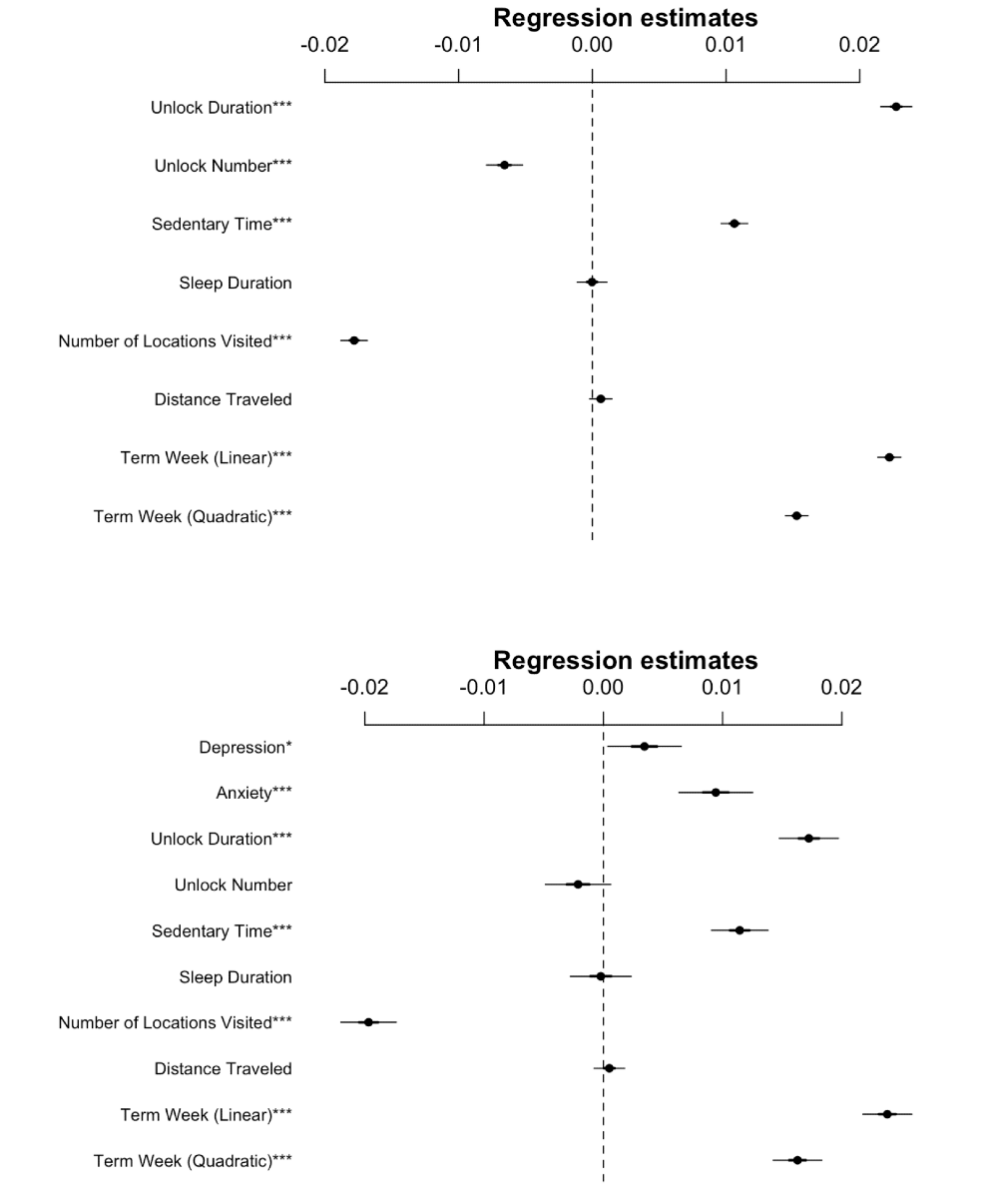
Coefficient plots from mixed linear models of COVID-19 news inferred with mobile smartphone features (top) or mobile smartphone features and self-reported mental health (bottom). Intercept and random intercepts per subject are not plotted. *, **, and ***: *P*<.05, <.01, and <.001, respectively. Where applicable, exact *P* values are shown in Table 3.

After establishing broad differences in sedentary time, depression, and anxiety between the Winter 2020 term and previous terms, the next goal was to determine if these behaviors changed in a finer-grained fashion, particularly mirroring the relative news coverage of COVID-19. The proportion of new stories including the term *coronavirus* remained at baseline until early January, with an unsurprisingly large increase appearing at the beginning of March (Figure 1). To ascertain which behaviors changed with increasing proportion of COVID-19 news reports, we included fixed effects for phone usage (unlock duration and unlock number), sedentary time, sleep duration, number of locations visited, and linear and quadratic academic term week regressors. Random intercepts per subject were included in the model. Each variable was scaled to aid convergence of the restricted maximum likelihood model and to obtain regression coefficients that could be compared for relative importance. All variables except sleep duration and distance traveled were significantly associated with the proportion of COVID-19 news reports (*P*<.001, Table 3 [left column], Figure 3 [top]). Phone usage (unlock duration) has the largest positive standardized coefficient, closely followed by the linear term week variable. The number of locations visited has the largest negative standardized coefficient.

In the second model of the COVID-19 news ratio, we again attempted to make inferences with mobile sensing features plus the addition of self-reported anxiety and depression scores. When anxiety and depression were added to the previously used sensing model, we observed that increased anxiety but not depression was significantly associated with a higher COVID-19 news ratio (*P*<.001, Table 3 [right column], Figure 3 [bottom]). The number of phone unlocks (unlock number) is significant in the first model but not in the second model. Since the standardized beta weights are relatively stable across all other variables, it appears that anxiety may have absorbed some of the variance associated with the unlock number in the first model; however, we cannot directly make this comparison due to the different subset of data in the second model. Again, we observed increased phone usage (unlock duration), increased sedentary time, and decreased number of locations visited, with standardized beta weights stable across both models. Both linear and quadratic term weeks were positively associated with the COVID-19 news ratio.

**Table 3:** Inferences of the proportion of COVID-19 news reports with smartphone features and self-reported mental health variables.

Variable	Dependent variables			
	COVID-19^a^ news model 1 (observations=100,300)		COVID-19 news model 2 (observations=18,432)	
	Parameter (SD)	*P* value	Parameter (SD)	*P* value
Depression	N/A^b^	N/A	0.003 (0.002)	.03
Anxiety	N/A	N/A	0.009 (0.002)	<.001
Unlock duration	0.023 (0.001)	<.001	0.017 (0.001)	<.001
Unlock number	–0.007 (0.001)	<.001	–0.002 (0.001)	.13
Sedentary time	0.011 (0.001)	<.001	0.011 (0.001)	<.001
Sleep duration	–0.00002 (0.001)	.97	–0.0002 (0.001)	.87
Number of locations visited	–0.018 (0.001)	<.001	–0.020 (0.001)	<.001
Distance traveled	0.001 (0.0004)	.14	0.001 (0.001)	.44
Term week (linear)	0.022 (0.0004)	<.001	0.024 (0.001)	<.001
Term week (quadratic)	0.015 (0.0004)	<.001	0.016 (0.001)	<.001
Constant	0.029 (0.002)	<.001	0.028 (0.002)	<.001

^a^COVID-19: coronavirus disease.

^b^Not applicable.

## Discussion

### Principal Findings

During the outbreak of a pandemic, the primary focus is on the pathogen and its influences on physical health. Mental health and behavioral changes are often considered secondary concerns. In the present study, we leveraged longitudinal data in a cohort of 217 college students to demonstrate the impact of the COVID-19 pandemic on mental health and behavior compared to previous academic terms. During the Winter 2020 academic term, sedentary time increased along with symptoms of anxiety and depression. Subsequent analyses examined the association between increased COVID-19 news coverage and behavior (inferred from mobile sensing data) and mental health. We found that with increasing COVID-19–related news, individuals were more sedentary, visited fewer locations (as inferred from GPS tracking), and showed increases in anxiety and depression. These analyses identified behavioral changes from smartphone sensing that are consistent with individuals adhering to the “Stay Safe, Stay Home” policies implemented by local and national governments.

During the Winter 2020 academic term, increased depression, anxiety, and sedentary time were observed, suggesting a large mental health and behavioral impact beyond the actual reach of the SARS-CoV-2 pathogen. The days and weeks leading up to and including the final examination period are a particularly stressful time for students [10]. We typically find that participants report relatively high scores of depression and anxiety during this time; however, these scores recover to baseline over the break [8]. The cyclical nature of college students’ mental health within a typical academic term provides a unique control in our study that might be otherwise difficult to untangle from periodic rises and falls in stress and anxiety during March as colleges across the United States conduct final examinations. Understanding behaviors during typical academic cycles can be fruitful for determining how everyday events impact students’ mental health and behaviors. Rare events such as the COVID-19 pandemic provide a unique opportunity to examine how mental health and behaviors deviate from baseline. To account for the cyclical nature of mental health throughout the academic term, we used linear and quadratic week of academic term variables, then looked for interactions and the main effects of the most recent (COVID-19–related) term. The increased depression, anxiety, and sedentary time, which were above and beyond what would normally be observed during a typical term, were attributed to the COVID-19 pandemic. Additionally, we did not observe a return to baseline for any of these 3 variables over the break, which stands in stark contrast to our previous study of this same cohort in prior academic terms. We did observe decreases in anxiety and depression that paralleled the typical post–final examination drop, which suggests some resilience in the face of COVID-19; however, the overall values remained elevated above those observed during typical academic breaks. This may also be consistent with the adjustment period to the rapidly changing landscape of social media, policies, and media coverage [25].

Individuals are generally more active over break periods; however, we observed a large increase in sedentary behavior, which can be attributed to the COVID-19 pandemic. Studies show that physical activity, particularly aerobic activity, reduces self-reported depressive symptoms with a similar efficacy to that of low-dose antidepressants [26]. Other studies have shown that increased sedentary time along with increased phone usage is implicated in depression and anxiety [12,27,28]. Taken as a whole, these findings suggest that during stay-at-home orders, individuals should increase physical activity and limit screen time in an attempt to lessen depressive symptoms.

As COVID-19 news reporting intensified, we observed increased sedentary behavior and duration of phone usage along with decreases in the number of locations visited and a decreased number of phone unlocks. Initially, the number of phone unlocks was somewhat surprisingly inversely associated with COVID-19 news; however, this is likely due to increased phone unlock duration (ie, screen time), which would otherwise span multiple phone unlocks. The decreased number of locations visited is consistent with the “Stay Home, Stay Safe” polices many governments have implemented; it also stands in contrast to initial work on social distancing among college students in the Netherlands, where social distancing policies were not implemented during the examined time period [9]. In the combined sensing and mental health model, the addition of depression and anxiety to the sensing model showed a strong inferential link between anxiety and COVID-19 news, while depression was marginally significant. In the combined model, the number of unlocks, sleep duration, and distance traveled were again not significant.

Primary takeaways from models inferring the proportion of COVID-19 news stories suggest that during the start of the COVID-19 pandemic, students were more depressed and anxious, used their phones more, visited fewer locations, and spent more time sedentary. At this critical time of increased depression and anxiety, we issue a call to public health officials and individual citizens to raise public awareness about the benefits of aerobic exercise and unplugging from technology (moderating phone usage), as each of these has previously been shown to have positive effects on alleviating anxiety and depression [8,26,28,29]. COVID-19 arrived locally during week 9 of the academic term. By the onset of the COVID-19 pandemic (academic term week 10), a significant deterioration in mental health and multiple behavioral changes were observed, which synchronizes with the implementation of rapid policy changes at the college, local, and national levels. These findings suggest a greatly expanded scope of the impact of the COVID-19 pandemic beyond the illness and deaths directly associated with the SARS-CoV-2 pathogen.

### Limitations and Future Directions

The current study has a variety of limitations, although most provide incentives for future research. First, our participants are approximately the same age, are undergraduate college students with smartphones compatible with the StudentLife app, and are willing to participate in a multiyear longitudinal research study; this limits the generalizability of the current findings to the general population. Simultaneously, it provides distinct advantages, such as longitudinal measurement across behavioral cycles, wherein previous academic terms can be compared to the term affected by COVID-19. A second limitation is the moderate number of individuals included in the study, with 217 total participants across all terms and 178 across the COVID-19 term (83%). Despite the moderate sample size, strong significant effects on mental health and behavior were observed, suggesting robust effects.

While smartphone sensing is quite robust, there are some limitations to the interpretation of the available data. When mobility is decreased, such as during a stay-at-home order, individuals may not have their mobile phones with them at all times, which could lead to overestimation of sedentary time. Additionally, participants may be preferentially accessing larger screens (eg, tablets or laptops); therefore, phone usage (as measured by screen unlock duration or number of unlocks) may underestimate the total amount of screen time. Even so, we observed increased phone usage despite the possibility of underestimated changes in the total amount of screen time. Future work should also identify the types of screens used during this increased consumption period and quantify the relative amounts of news, social media, and other content consumed. In further work, we could also use smartwatches to improve the measurement of behaviors such as sedentary time and allow for more frequent sampling of phone usage, location, and other measures.

While this work primarily focuses on the initial days of the COVID-19 pandemic, future work would be well suited to investigating differences in mental health and behaviors between typical residential academic terms and terms that have been shifted from residential to online coursework due to COVID-19. Furthermore, identifying causal patterns between the pandemic, policy changes on national, local, and college levels, mental health, and behavior could provide further insight into the optimal development of interventions designed to mitigate mental health crises in the face of global crises. These findings suggest a greatly expanded scope of the impact of the COVID-19 pandemic beyond that directly associated with the SARS-CoV-2 pathogen.

### Conclusions

Understanding behaviors during typical academic cycles can be fruitful for determining how everyday events impact students’ mental health and behaviors. Fringe events such as the COVID-19 pandemic provide opportunities to examine how mental health and behaviors deviate from baseline.

This study provides preliminary insight into mental health and related behaviors during the initial phases of the COVID-19 pandemic. Depression, anxiety, and sedentary time increased as the COVID-19 pandemic encroached on a college campus in parallel with large-scale policy changes. Using a mixed linear model of smartphone mobile sensing and self-reported mental health questions, we were able to infer the proportion of COVID-19–related news stories; moreover, we could validate that participants’ mental health and related behaviors changed in lockstep with increased media coverage and proximity of the pandemic. For these college students, the early days of the pandemic coincided with what is typically a time of increased time and depression, and we observed altered behavioral patterns and a decrease in mental health above and beyond typical academic terms. Much more work remains to be done to understand how behaviors and mental health change and interact in the face of monumental adverse global events.

## Abbreviations

COVID-19: coronavirus disease
DBSCAN: density-based spatial clustering of applications with noise
EMA: ecological momentary assessment
GAD-2: Generalized Anxiety Disorder-2
PHQ-2: Patient Health Questionnaire-2
PHQ-4: Patient Health Questionnaire-4
SARS-CoV-2: severe acute respiratory syndrome coronavirus 2
WHO: World Health Organization
